# Compressed SENSitivity Encoding (SENSE): Qualitative and Quantitative Analysis

**DOI:** 10.3390/diagnostics14151693

**Published:** 2024-08-05

**Authors:** Eliseo Picchi, Silvia Minosse, Noemi Pucci, Francesca Di Pietro, Maria Lina Serio, Valentina Ferrazzoli, Valerio Da Ros, Raffaella Giocondo, Francesco Garaci, Francesca Di Giuliano

**Affiliations:** 1 Department of System Medicine, University of Rome Tor Vergata, Via Montpellier 1, 00133 Rome, Italy; eliseo.picchi@uniroma2.it; 2 Diagnostic Imaging Unit, University Hospital Tor Vergata, Viale Oxford 81, 00133 Rome, Italy; silvia.minosse2@gmail.com (S.M.); noemi.pucci@students.uniroma2.eu (N.P.); francesca.dipietro@hotmail.com (F.D.P.); marialinaserio@gmail.com (M.L.S.); valerio.da.ros@uniroma2.it (V.D.R.); francesco.garaci@uniroma2.it (F.G.); 3 Department of Biomedicine and Prevention, University of Rome Tor Vergata, Viale Montpellier 1, 00133 Rome, Italy; valentina.ferrazzoli@alumni.uniroma2.eu (V.F.); raffaella.giocondo@uniroma2.it (R.G.); 4 Neuroradiology Unit, University Hospital Tor Vergata, Viale Oxford 81, 00133 Rome, Italy

**Keywords:** compressed SENSE, compressed sensing-sensitivity encoding (compressed-SENSE), MRI

## Abstract

Background. This study aimed to qualitatively and quantitatively evaluate T1-TSE, T2-TSE and 3D FLAIR sequences obtained with and without Compressed-SENSE technique by assessing the contrast (C), the contrast-to-noise ratio (CNR) and the signal-to-noise ratio (SNR). Methods. A total of 142 MRI images were acquired: 69 with Compressed-SENSE and 73 without Compressed-SENSE. All the MRI images were contoured, spatially aligned and co-registered using 3D Slicer Software. Two radiologists manually drew 12 regions of interests on three different structures of CNS: white matter (WM), grey matter (GM) and cerebrospinal fluid (CSF). Results. C values were significantly higher in Compressed-SENSE T1-TSE compared to No Compressed-SENSE T1-TSE for three different structures of the CNS. C values were also significantly lower for Compressed-SENSE 3D FLAIR and Compressed-SENSE T2-TSE compared to the corresponding No Compressed-SENSE scans. While CNR values did not significantly differ in GM-WM between Compressed-SENSE and No Compressed-SENSE for the 3D FLAIR and T1-TSE sequences, the differences in GM-CSF and WM-CSF were always statistically significant. Conclusion. Compressed-SENSE for 3D T2 FLAIR, T1w and T2w sequences enables faster MRI acquisition, reducing scan time and maintaining equivalent image quality. Compressed-SENSE is very useful in specific medical conditions where lower SAR levels are required without sacrificing the acquisition of helpful diagnostic sequences.

## 1. Introduction

Magnetic resonance imaging (MRI) is the primary non-invasive radiological method for evaluating a wide range of illnesses. However, it is still a time-consuming procedure that causes discomfort to patients, in particular to those suffering from neurological disorders and claustrophobia, as well as to paediatric patients who may not bear long time acquisition; moreover, the reduction in the acquisition time can also be considered an important goal in patients with MR-conditional implants. 

Several MRI techniques have been developed to reduce acquisition times, ranging from accelerated sequences to reconstruction methods [1]. The parallel imaging is a robust method, allowing to significantly reduce the scan time without affecting the image quality thanks to the properties of the new coil arrays [2,3,4]; nowadays, the most common parallel imaging techniques are the SENSitivity Encoding-SENSE, Compressing Sense-CS, GeneRalized Autocalibrating Partial Parallel Acquisition—GRAPPA and the Iterative self-consistent parallel imaging reconstruction (SPIRiT) offered by the main MR vendors.

Although CS can reduce scan time by randomly under-sampling data, SENSE evenly under-samples data and uses coil sensitivity information to restore the entire image. The SENSE technique is a method for parallel imaging that uniformly under-samples data and uses coil sensitivity information to restore the full image. On the other hand, CS allows for scan time reduction by randomly under-sampling data compared to other parallel imaging techniques. The sampling in CS is described as “variable density incoherently under-sampled”, where in the centre of k-space is more densely sampled to account for the fact that most of the contrast information comes from this region [5]. To improve the coherence of the sampling scheme, a sparsity-enforcing (iterative) reconstruction should be employed to reduce the pseudo-noise introduced by the unconventional sampling process. This can be achieved using a dedicated cost function for model-based reconstruction [6].

Currently, SENSE and CS techniques can be combined to obtain images with acquisition acceleration factors far superior to those achievable with parallel or CS techniques alone [7]. One such technique, called compressed sensing-sensitivity encoding (Compressed-SENSE), is provided by Philips Healthcare (Philips Healthcare, Amsterdam, The Netherlands).

However, to determine the extent to which acquisition can be accelerated without compromising image quality, quantitative and qualitative analyses are required. These analyses will determine if the diagnostic power would differ when using Compressed-SENSE and if any new types of artefacts would be observed.

The aim of this study was to compare qualitative and quantitative imaging techniques, specifically two-dimensional (2D) axial T1 Turbo Spin Echo-TSE, 2D axial T2-TSE and 3D Fluid Attenuated Inversion Recovery (FLAIR) with and without the Compressed-SENSE technique in healthy volunteers to avoid bias in the analysis related to specific neurological diseases. The contrast (C), contrast-to-noise ratio (CNR) and signal-to-noise ratio (SNR) were assessed.

## 2. Methods

### 2.1. Population

This retrospective study included brain MRI scans of 142 healthy subjects, 69 of which were acquired with Compressed-SENSE and 73 without Compressed-SENSE. The Compressed-SENSE group consisted of 31 men and 38 women (aged 19–86 years), while the No Compressed-SENSE group consisted of 36 men and 37 women (aged 13–84 years). The inclusion criteria were healthy volunteers, aged between 10 and 90 years without gender, race, language and educational predilection. The exclusion criteria for the enrolled subjects were history of head trauma, stroke, epilepsy, CNS infections, demyelinating disease, previous or active neurological diseases, tumours, coinfections, illicit substance abuse in five years and any kind of contraindication to MRI examinations.

The study was approved by the Ethics Committee of the University Hospital and Faculty of Medicine of Tor Vergata University of Rome. The authors confirm that all imaging methods were employed in accordance with national and institutional guidelines and regulations, in accordance with the 1964 Declaration of Helsinki and its subsequent amendments.

Informed written consent was obtained from all the patients (or their parents in the case of minors). 

Sample size of 104 people was assessed according to Z formula and a confidence interval of 95% with 80% power to detect any significant difference between the compressed and non-compressed sequences with a significance level of 0.05.

### 2.2. Protocol Optimisation

Two neuroradiologists and an MRI physicist agreed to optimise the compressed sense factor (CS factor) in order to decrease examination time or improve image resolution, and thus included Compressed-SENSE in the clinical brain MR procedure. To guarantee that the final images were the same as those produced without Compressed-SENSE, the CS factor was carefully selected for each sequence. This method was applied to FLAIR and TSE sequences. The sequence was scanned with the CS factor while the denoising level was varied. This method was then used to figure out the denoising level.

### 2.3. MRI Protocol

MRI examinations were performed on a 3T scanner system (Achieva d-STREAM, Philips Medical Systems, Best, The Netherlands) with a dedicated 32-channel head coil.

The MRI protocol in both groups included the following: (a) axial T2-weighted (w) TSE sequence; (b) axial T1-w TSE sequence; (c) 3D T2-w FLAIR (T2 3D FLAIR) sequence; (d) echo planar diffusion weighted imaging (DWI); (e) axial T2 fast field echo (FFE) sequence. Compressed-SENSE was used to accelerate T2w TSE, T1w TSE, T2 3D FLAIR sequences only in Compressed-SENSE group.

Table 1 reports the specific acquisition parameters for the sequences that were evaluated.

**Figure 1:**
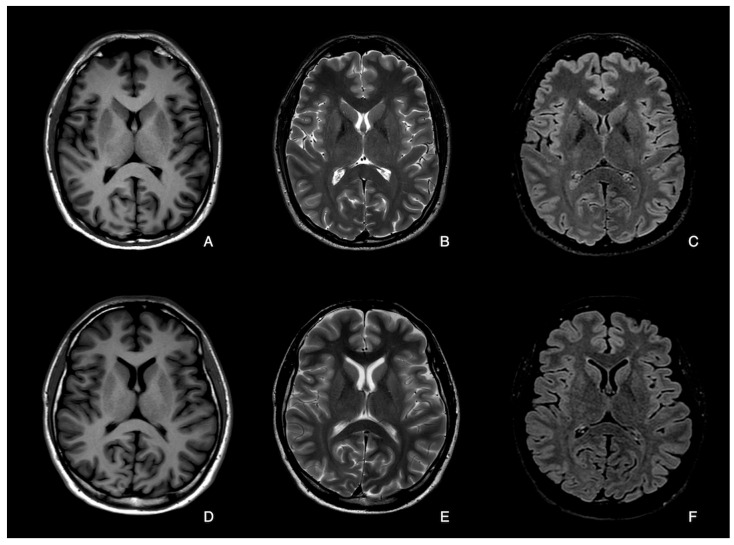
Comparison between Compressed-SENSE and No Compressed-SENSE sequences in two healthy subjects, both 28 years old. Upper panel: Compressed-SENSE T1-Turbo Spin Echo (TSE) (**A**), Compressed-SENSE T2-TSE (**B**) and Compressed-SENSE 3D-T2-FLAIR (**C**). Lower panel: T1-TSE (**D**), T2-TSE (**E**) and 3D-T2-FLAIR (**F**).

Both quantitative and qualitative methods were used to analyse the Compressed-SENSE and conventional 3D FLAIR, 2D T2 TSE, and T1 IR TSE axial brain sequences obtained at 3T (Figure 1).

### 2.4. Qualitative Image Analysis

Two double-blind radiologists, with more than 10 years and 3 years of neuroimaging experience, respectively, evaluated the qualitative analysis of Compressed-SENSE and No Compressed-SENSE images (T1w, T2w, and T2-FLAIR). The scoring system used for the analysis was as follows:

- Score 5: Excellent; acceptable for diagnostic use, complete absence of artefacts;
- Score 4: Good; acceptable for diagnostic use (only minor artefacts);
- Score 3: Fair; acceptable for diagnostic use but with minor issues;
- Score 2: Sufficient; acceptable for diagnostic use but severely mixed with the background;
- Score 1: Insufficient; not acceptable for diagnostic use.

The qualitative score was obtained considering the following criteria: (i) demarcation of caudate head and sulci, (ii) grey-white matter differentiation (at the level of the lateral ventricles), (iii) artefacts in the posterior cranial fossa, (iv) motion artefacts, (v) low image resolution artefacts, (vi) low SNR, (vii) phase encoding, (viii) parallel imaging artefacts, (ix) flow pulsation artefacts in the ventricles in accordance with previous published papers [8,9,10].

### 2.5. Quantitative Image Analysis

The Compressed-SENSE and No Compressed-SENSE T1w, T2w e 3D-FLAIR were visualised, contoured and opportunely spatially aligned and co-registered using (3D) Slicer Software (Release 4.6.2). Two radiologists, in consensus, manually drew twelve regions of interests (ROIs), with a diameter of 4 mm, on three different structures of the central nervous system (CNS): (i) white matter (WM), (ii) grey matter (GM), and (iii) cerebrospinal fluid (CSF) as reported by Di Giuliano et al. [11,12].

For the WM, the ROIs were positioned in the (i) left and right centrum semiovale and (ii) genu and splenium of corpus callosum; for the GM, the ROIs were positioned in the (i) left and right frontal and occipital cortex and (ii) thalami; for CSF, the ROIs were positioned in the left and right anterior horn of the lateral ventricles. The mean and the standard deviation of the signal intensity were calculated in each ROI.

The quantitative analysis of the images was performed by evaluating C, CNR and SNR, using the following equation:

$$C=\frac{I_{i}-I_{j}}{I_{i}+I_{j}}$$

$$CNR=\frac{I_{i}-I_{j}}{\sigma_{median}^{12}}$$

$$SNR=\frac{I_{i}}{\sigma_{i}}$$

where *I_i_* and *I_j_* are the mean signal intensity in two different tissue type; $\sigma_{median}^{12}$ is the median standard deviation of all 12 ROIs and $\sigma_{i}\;$is the standard deviation for *i*th ROI.

Patients were combined for C, CNR and SNR.

The ROIs were loaded in MATLAB Version 9.7.0, Release 2019b, where dedicated scripts for quantitative image analysis was developed.

### 2.6. Statistical Analysis

The weighted Kappa test was performed to evaluate the inter-rater agreement between the two radiologists for Compressed-SENSE and conventional T1, T2 and 3D-FLAIR sequences: a Kappa value > 0.81 was considered very good, a value between 0.61 and 0.80 was considered good, 0.41–0.60 was considered moderate, 0.21–0.40 was considered fair and a value < 0.20 was considered poor.

Because the sample size was fewer than 50, the Shapiro–Wilk normality test was used to assess the variables’ normal distribution. The variables in the study were non-normally distributed; therefore, the median and 95% confidence intervals (95% CIs) were used for all the analysed variables. 

Data are expressed as median and 95% CIs. *p*-values refer to the Mann–Whitney U test.

## 3. Results

### 3.1. Qualitative Image Analysis

The image quality scores for Compressed-SENSE and No Compressed-SENSE T1w, T2w and 3D-FLAIR are reported in Table 2.

The image quality scores obtained by two readers were higher for the No Compressed-SENSE and Compressed-SENSE T1-TSE; the mean differences between the No Compressed-SENSE T1W and Compressed-SENSE T1W images quality scores for reader 1 and reader 2 were 0.02 and 0.06, respectively.

The mean differences between the No Compressed-SENSE 3D FLAIR and Compressed-SENSE 3D FLAIR image quality scores for reader 1 and reader 2 were 0.11 and 0.81, respectively.

Conversely, the mean differences between the No Compressed-SENSE and Compressed-SENSE T2-TSE image quality scores were −0.11 for reader 1 and −0.07 for reader 2.

The weighed Kappa values for the agreement between readers was good (0.66) for No Compressed-SENSE T2W images, while for other images it was moderate: 0.45 for No Compressed-SENSE T1W, 0.60 for No Compressed-SENSE 3D-FLAIR, 0.41 for Compressed-SENSE T1W and Compressed-SENSE T2W and 0.54 for Compressed-SENSE 3D FLAIR.

### 3.2. Quantitative Image Analysis

The C, CNR and SNR values derived from 3D FLAIR, T2-TSE and T1-TSE with and without Compressed-SENSE sequences are illustrated in Table 3.

The C values were statistically significant for the three different structures of CNS (*p* < 0.05) being higher for Compressed-SENSE T1-TSE compared to No Compressed-SENSE ones. The C values were statistically significant for the GM-CSF and WM-CSF (*p* < 0.05) and lower for Compressed-SENSE 3D FLAIR and Compressed-SENSE T2-TSE compared to corresponding No C-SENSE scans.

The CNR values did not significantly differ in GM-WM between Compressed-SENSE and No Compressed-SENSE for the 3D FLAIR and T1-TSE sequences while the differences in GM-CSF and WM-CSF were always statistically significant (*p* < 0.05) as reported in Table 4. 

The differences in SNR between the Compressed-SENSE and No Compressed-SENSE T2-TSE sequences were statistically significant for all the CNS structures except for CSF: specifically, No Compressed-SENSE T2-TSE sequence showed higher ROIs values than C-SENSE T2-TSE one.

The SNR was statistically significant for CSF being higher in CS 3D FLAIR sequence compared to No Compressed-SENSE one. The SNR values were statistically significant higher for the Compressed-SENSE T1-TSE sequences in the frontal and occipital GM compared to No Compressed-SENSE TSE ones; furthermore, even though the SNR for the Compressed-SENSE T1-TSE sequence was higher in several brain structures compared to No Compressed-SENSE T1-TSE, no statistically significant differences were detected as reported in Table 5.

The 3D FLAIR detected statistically significant differences between Compressed-SENSE and No Compressed-SENSE sequences only in the CSF with higher values for the Compressed-SENSE sequence than No Compressed-SENSE ones. 

### 3.3. Subgroup Qualitative and Quantitative Image Analysis

We also performed a subgroup analysis by dividing our enrolled population into three groups: group 1 (with ages ranged between 13 and 37 years), group 2 (with ages ranged between 38 and 62 years) and group 3 (with ages ranged between 63 and 86 years); the results of qualitative and quantitative image analysis in these subgroups are reported in Supplementary Materials (Tables S1–S10). The quantitative image analysis did not show significant differences between subgroups. The qualitative image analysis predominantly demonstrated moderate agreement between readers in the subgroups analysis except for the Compressed-SENSE T1-TSE in groups 2 and 3.

## 4. Discussion

Compressed-SENSE is a strategy that accelerates MRI scans while preserving imaging quality for longer sequences, such as mDIXON, 3D BRAIN VIEW, and susceptibility-weighted imaging-phase (SWI). This is particularly useful for non-compliant and monitored patients. Additionally, Compressed-SENSE has been shown to decrease the Specific Absorption Rate (SAR) in long-term tests. 

Few clinical studies have evaluated the performance of C-SENSE in body, brain and the cranial nerve imaging [13,14,15,16,17,18,19,20,21,22].

Qualitative analysis revealed a higher median value for the Compressed-SENSE T2w images compared to the No Compressed-SENSE T2w images with good agreement between observers. This finding is in line with Meister et al. [19], who suggested that the slightly more homogeneous signal of GM and WM on Compressed-SENSE images, as opposed to standard T2-TSE images, may be due to the denoising capability of Compressed-SENSE [4,5,22,23]. However, Monch reported lower image quality for the T2-TSE sequence than for the Compressed-SENSE T2-TSE sequence, although the difference was not statistically significant [24].

In contrast, the quality of Compressed-SENSE T1w and Compressed-SENSE 3D FLAIR was lower than that of non-Compressed-SENSE ones, with moderate inter-reader agreement. These results contradict the literature [7,17], which showed that accelerated Compressed-SENSE 3D FLAIR imaging provides equivalent image quality compared to corresponding conventional imaging. 

Molnar et al. demonstrated a statistically significant negative correlation between the Compressed-SENSE acceleration factor and the evaluation score for corticomedullary differentiation, sulcus delineation, and noise in Compressed-SENSE T2 TSE axial scans. However, there was no statistically significant difference in the diagnostic quality of Compressed-SENSE T2-TSE images acquired with Compressed-SENSE factors ranging between 2 and 3 and those acquired without Compressed-SENSE [25].

The Compressed-SENSE T1-TSE demonstrated significantly higher contrast values than the No Compressed-SENSE T1-TSE in the three different brain structures. The statistical analysis revealed that the No Compressed-SENSE T2-TSE and No Compressed-SENSE T2-3D-FLAIR sequences had significantly higher values than the Compressed-SENSE sequences, except for GM-WM, where no statistically significant differences were found. These results may be attributed to the intrinsic high tissue contrast, which could be reduced by the strong Compressed-SENSE denoising applied to the T2 weighted sequences. Additionally, the Compressed-SENSE T1-TSE sequence appears to be less sensitive to denoising.

Furthermore, the No Compressed-SENSE T2-TSE sequence exhibited significantly higher CNR values than the Compressed-SENSE T2-TSE sequence for the three brain structures, enabling better identification of brain lesions. The CNR did not differ significantly between the Compressed-SENSE and No Compressed-SENSE 3D FLAIR and T1-TSE sequences in GM-WM.

Even though TR and TE were the same for the T1-TSE No Compressed-SENSE and T1-TSE Compressed-SENSE, the TR values between Compressed-SENSE and No Compressed-SENSE T2-TSE and T2-3D-FLAIR were different: in particular, the preset TR values were higher for the T2 Compressed-SENSE sequences than the No Compressed-SENSE ones, and this seems to be related to the need to have more signal from the Compressed sequences due to the intrinsic under-sampling technique. Moreover, to gain signal, we increased the number of averages (NSA) for the Compressed-SENSE T2-TSE. 

Although the contrast and CNR values were lower for the Compressed-SENSE T2-TSE and Compressed-SENSE T2-3D FLAIR sequences, as well as for the SNR values of the Compressed-SENSE T2-TSE, these did not affect the diagnostic ability of the Compressed-SENSE sequences.

Moreover, we are well aware that the higher default TR values for the Compressed-SENSE T2-TSE and Compressed-SENSE T2-3D-FLAIR than No-Compressed ones had a strong impact on time. In our opinion, the conjunction between the increase of NSA for the C-SENSE T2-TSE and TR values determined the increase in acquisition time and therefore a similar total scan time between the Compressed and No-Compressed T2 sequences. Nevertheless, in our opinion, a small reduction in the TR value for the C-SENSE T2 sequences could lead to time reduction without a significant impact on image quality.

The SNR values for the No Compressed-SENSE T2-TSE were higher than those for the Compressed-SENSE T2-TSE, except for the CSF. Our SNR values for the Compressed-SENSE T2-TSE were lower than the conventional values in all brain regions (*p* < 0.05), including the basal ganglia. This may be related to the denoising level, which disagrees with Monch et al. [24]. However, noise measurement might be difficult when using parallel imaging technology due to the spatial nonuniformity of the noise as well as the exacerbation of Rician bias at lower signal levels; even though we evaluated the SNR and CNR through the standard deviation of the contoured regions, further approaches are suggested in the literature [26,27,28]. 

Our analysis revealed that higher acceleration factors were achieved for 3D scans compared to the 2D scans due to more room for aggressive under-sampling. This finding is consistent with Sartoretti et al. [29], who observed a 32% reduction in sequence acquisition time while maintaining the same image quality using a C-SENSE factor of 7.8 for T2 3D FLAIR. We set the Compressed-SENSE factor at 9 with an acceleration of 17% for the same sequence, with no statistically significant difference in image quality.

Duan et al. [30] evaluated the Compressed-SENSE method for 3D T1w turbo field echo (TFE) brain imaging. They compared it to the previous studies of 3D T1 [18,29,31] and T2-FLAIR images. The authors evaluated more acceleration factors [7,29,31,32,33] compared to both the traditional acceleration technique (SENSE) and the non-accelerated imaging [18,24]. The study demonstrated a significant decrease in SNR and CNR in all accelerated sequences, with CS factor 3 showing better performance in terms of image artefacts due to its shorter scan time, indicating that it can be recommended as the optimal acceleration factor [30]. 

Sartoretti et al. [34] demonstrated that the application of Compressed-SENSE led to an increase in motion artefacts with a higher spatial frequency. Additionally, images obtained with C-SENSE may exhibit specific artefacts, such as streaky-linear, wax-layer and starry-sky. In our study, we found ‘streaky-linear’ type A artefacts in 36% of patients in Compressed-SENSE 3D-FLAIR sequences. These artefacts were observed particularly in subjects with major motion artefacts, as reported by Sartoretti [34]. Type A streaks, which were either long or short lines, appeared centrally and peripherally on the image in a horizontal or oblique arrangement. Sartoretti et al. [34] state that type-A streaks can always be provoked on transverse, coronal and sagittal images if the reconstruction voxel is smaller than the acquisition voxel. Although our reconstruction voxel was the same as the acquisition voxel (both 1 mm), we also observed these types of artefacts.

Furthermore, in Compressed-SENSE 3D-FLAIR sequences, type B artefacts with a “streaky-linear” appearance were found in 16% of patients, while only one patient presented the ‘starry-sky’ artefacts, in which some structures appeared slightly pixelated and grainy. These results are in line with those observed by Sartoretti [34]. The sequences with the highest Compressed-SENSE factor exhibited the most artefacts. The Compressed-SENSE factor used was 8.2 for 3D FLAIR, while ours was 9. In particular, we felt that the streaky artefacts had little impact on image quality and did not affect image evaluation, given their regular occurrence as described in the literature [35,36], although they may influence image interpretation if they remain undetected by image readers due to their unpredictable, often not easily recognisable and unfamiliar nature [34].

Attention must be paid to the setting of the optimal denoising level for the Compressed-SENSE sequences; the aim of denoising is to minimise the amount of noise in the images due to unwanted signals that compromise the quality of a desired signal. The level of denoising determines how much noise is reduced or balanced: images with high denoising are smoother and less noisy, whereas images with weak denoising are sharper but noisier. Thereby strong denoising should be used with caution, as it can blur the white-grey matter interface. 

This study had several limitations. A major limitation is that qualitative and quantitative image analysis of Compressed-SENSE and No Compressed-SENSE sequences were not acquired and compared in the same patients. Secondly, we did not assess the effect of Compressed-SENSE acceleration on selected sequences in the presence of different pathologies, or how it would affect the diagnostic task of their detection/characterisation: efforts should also be made to extend the evaluation of Compressed-SENSE sequences in different brain diseases with larger human datasets. 

## 5. Conclusions

Our quantitative and qualitative analysis confirmed the potential of Compressed-SENSE for 3D T2 FLAIR, T1w and T2w sequences in the neuroimaging field, where this acceleration technique allows the scan time to be reduced while maintaining virtually equivalent image quality; furthermore, the Compressed-SENSE technique appears to be very useful in specific medical conditions (e.g., implants) where lower SAR levels are required, which can be achieved without sacrificing the acquisition of other useful diagnostic sequences.

## Figures and Tables

**Table 1:** MRI acquisition parameters.

	Compressed-SENSE			No Compressed-SENSE		
	T1-TSE	T2-TSE	3D T2-FLAIR	T1-TSE	T2-TSE	3D T2-FLAIR
Acquisition matrix	308 × 257	420 × 322	252 × 251	308 × 226	420 × 350	228 × 228
Field of view (cm)	23	23	25	23	23	25
Repetition time (ms)	2000	6200	6000	2000	3000	4800
Echo time (ms)	20	90	340	20	80	280
Slice thickness (mm)	3	1.5	1	4	4	1.1
Intersection gap (mm)	1	1	−0.5	1	1	−0.55
Number of averages	1	2	1	1	1	2
Bandwidth (kHz)	165.7	217.2	318.7	169.8	195.8	1166.5
C-SENSE factor	3	2	9	-	-	-
Acquisition time	2′34″	3′08″	3′50″	3′00″	2′42″	4′34″

TSE: Turbo Spin Echo; FLAIR: Fluid Attenuated Inversion Recovery.

**Table 2:** Summary statistics of qualitative image analysis.

Sequences	Reader 1	Reader 2
T1-TSE Compressed-SENSE	4.93 [4–5]	4.78 [3–5]
T1-TSE No Compressed-SENSE	4.95 [4–5]	4.84 [4–5]
T2-TSE Compressed-SENSE	4.93 [4–5]	4.77 [4–5]
T2-TSE No Compressed-SENSE	4.82 [4–5]	4.70 [4–5]
3D T2 FLAIR Compressed-SENSE	4.78 [4–5]	3.97 [3–5]
3D T2 FLAIR No Compressed-SENSE	4.89 [4–5]	4.78 [4–5]

TSE: Turbo Spin Echo; FLAIR: Fluid Attenuated Inversion Recovery. Data are expressed as average (outside square brackets) and range (inside square brackets).

**Table 3:** Summary statistics of contrast (C).

		Compressed-SENSE			No Compressed-SENSE			
		Median	25th	75th	Median	25th	75th	*p*-Value
FLAIR	GM-WM	0.09	0	0.17	0.08	0.01	0.15	0.130
	GM-CSF	0.64	0.56	0.69	0.77	0.71	0.82	<0.001 *
	WM-CSF	0.58	0.51	0.64	0.74	0.66	0.79	<0.001 *
T1	GM-WM	−0.17	−0.24	−0.13	−0.19	−0.25	−0.13	0.009 *
	GM-CSF	0.68	0.64	0.71	0.65	0.59	0.7	<0.001 *
	WM-CSF	0.76	0.74	0.79	0.75	0.71	0.77	<0.001 *
T2	GM-WM	0.11	0.05	0.17	0.1	0.05	0.16	0.849
	GM-CSF	−0.52	−0.55	−0.47	−0.39	−0.43	−0.33	<0.001 *
	WM-CSF	−0.59	−0.62	−0.56	−0.48	−0.51	−0.44	<0.001 *

Data are expressed as median, 25th and 75th percentile. *p*-values refer to the Wilcoxon test. Statistically significant correlations for * *p* < 0.05. White matter (WM), grey matter (GM) and cerebrospinal fluid (CSF).

**Table 4:** Summary statistics of contrast-to-noise ratio (CNR).

		Compressed-SENSE			No Compressed-SENSE			
		Median	25th	75th	Median	25th	75th	*p*-Value
FLAIR	GM-WM	2.32	0.09	4.73	1.95	0.33	3.99	0.150
	GM-CSF	11.38	8.81	14.51	12.82	10.10	15.46	0.002 *
	WM-CSF	9.05	7.00	11.68	10.66	8.30	12.93	<0.001 *
T1	GM-WM	−9.03	−11.99	−6.38	−8.79	−11.94	−6.22	0.633
	GM-CSF	17.05	13.85	20.63	15.03	12.03	18.65	<0.001 *
	WM-CSF	25.71	22.37	31.31	24.69	19.30	29.24	0.007 *
T2	GM-WM	3.85	1.59	6.29	4.72	2.07	8.07	<0.001 *
	GM-CSF	−43.52	−52.04	−35.44	−33.35	−40.00	−27.02	<0.001 *
	WM-CSF	−47.30	−57.74	−39.84	−38.82	−45.99	−31.38	<0.001 *

Data are expressed as median, 25th and 75th percentile. *p*-values refer to the Wilcoxon test. Statistically significant correlations for * *p* < 0.05. White matter (WM), grey matter (GM) and cerebrospinal fluid (CSF).

**Table 5:** Summary statistics of signal-to-noise ratio (SNR).

		Compressed-SENSE			No Compressed-SENSE			
		Median	25th	75th	Median	25th	75th	*p*-Value
FLAIR	FC	18.45	15.48	21.70	18.08	14.91	20.24	0.207
	Ge	11.64	9.63	13.46	12.40	9.85	13.74	0.235
	CSF	3.26	2.90	3.81	1.90	1.51	2.39	<0.001 *
	Sp	10.73	9.08	13.65	11.39	9.26	13.04	0.797
	CS	14.93	13.10	18.29	15.17	12.44	17.47	0.540
	OC	14.02	11.95	16.73	14.62	12.13	16.43	0.803
	Th	12.32	10.95	15.94	12.99	11.21	15.38	0.841
T1	FC	18.83	14.40	21.91	16.51	13.63	19.49	0.025 *
	Ge	29.91	23.44	36.06	28.48	23.01	33.30	0.269
	CSF	4.00	3.44	4.87	4.21	3.62	5.00	0.331
	Sp	29.91	25.83	35.78	29.16	23.77	34.13	0.232
	CS	30.68	26.07	36.10	28.39	23.63	32.54	0.084
	OC	21.19	17.73	25.15	19.48	16.84	22.67	0.028 *
	Th	23.69	20.56	28.08	22.27	18.82	26.44	0.201
T2	FC	23.45	20.07	27.88	30.32	27.07	36.43	<0.001 *
	Ge	15.76	13.46	17.78	20.57	17.36	23.45	<0.001 *
	CSF	64.21	53.73	77.68	61.58	51.03	71.94	0.073
	Sp	15.97	13.12	18.84	20.29	16.16	23.71	<0.001 *
	CS	18.53	16.22	21.69	23.59	20.86	28.34	<0.001 *
	OC	18.39	15.42	21.01	24.05	19.91	27.95	<0.001 *
	Th	20.96	17.25	23.73	25.53	22.33	29.46	<0.001 *

Data are expressed as median, 25th and 75th percentile. *p*-values refer to the Wilcoxon test. Statistically significant correlations for * *p* < 0.05. Frontal cortex (FC), genu of corpus callosum (Ge), cerebrospinal fluid (CSF), splenium of corpus callosum (Sp), centrum semiovale (CS), occipital cortex (OC), thalami (Th).

## Data Availability

The original contributions presented in the study are included in the article/Supplementary Material, further inquiries can be directed to the corresponding author.

## Supplementary Materials

The following supporting information can be downloaded at: https://www.mdpi.com/article/10.3390/diagnostics14151693/s1, Table S1. Qualitative subgroups analysis; Table S2. Summary statistics of Contrast (C) of Group 1 (age range 13–37); Table S3. Summary statistics of Contrast-to-Noise Ratio (CNR) of Group 1 (age range 13-37); Table S4. Summary statistics of Signal-to-Noise Ratio (SNR) of Group 1 (age range 13–37); Table S5. Summary statistics of Contrast (C) of Group 2 (age range 38–62); Table S6. Summary statistics of Contrast-to-Noise Ratio (CNR) of Group 2 (age range 38–62); Table S7. Summary statistics of Signal-to-Noise Ratio (SNR) of Group 2 (age range 38–62); Table S8. Summary statistics of Contrast (C) of Group 3 (age range 63–86); Table S9. Summary statistics of Contrast-to-Noise Ratio (CNR) of Group 3 (age range 63–86); Table S10. Summary statistics of Signal-to-Noise Ratio (SNR) of Group 3 (age range 63–86).

## Abbreviations

FLAIR	Fluid Attenuated Inversion Recovery
C-SENSE	Compressed SENsing–Sensitivity Encoding
C	contrast
CNR	contrast-to-noise ratio
SNR	signal-to-noise ratio
MRI	magnetic resonance imaging
CNS	central nervous system
WM	white matter
GM	grey matter
CSF	cerebrospinal fluid
TSE	Turbo Spin Echo
SENSE	sensitivity encoding
CS	Compressed Sensing
ROI	Regions Of Interest
SWI	Susceptibility Weighted Imaging-phase
SAR	Specific Absorption Rate
TFE	Turbo Field Echo

## References

[1] Tsao J., Kozerke S. (2012). MRI Temporal Acceleration Techniques. J. Magn. Reson. Imaging.

[2] Hamilton J., Franson D., Seiberlich N. (2017). Recent Advances in Parallel Imaging for MRI. Prog. Nucl. Magn. Reson. Spectrosc..

[3] Pruessmann K.P., Weiger M., Scheidegger M.B., Boesiger P. (1999). SENSE: Sensitivity Encoding for Fast MRI. Magn. Reson. Med..

[4] Liang D., Liu B., Wang J., Ying L. (2009). Accelerating SENSE Using Compressed Sensing. Magn. Reson. Med..

[5] Lustig M., Donoho D., Pauly J.M. (2007). Sparse MRI: The Application of Compressed Sensing for Rapid MR Imaging. Magn. Reson. Med..

[6] Fessler J. (2010). Model-Based Image Reconstruction for MRI. IEEE Signal Process. Mag..

[7] Toledano-Massiah S., Sayadi A., de Boer R., Gelderblom J., Mahdjoub R., Gerber S., Zuber M., Zins M., Hodel J. (2018). Accuracy of the Compressed Sensing Accelerated 3D-FLAIR Sequence for the Detection of MS Plaques at 3T. Am. J. Neuroradiol..

[8] Granberg T., Uppman M., Hashim F., Cananau C., Nordin L.E., Shams S., Berglund J., Forslin Y., Aspelin P., Fredrikson S. (2016). Clinical Feasibility of Synthetic MRI in Multiple Sclerosis: A Diagnostic and Volumetric Validation Study. Am. J. Neuroradiol..

[9] Blystad I., Warntjes J.B.M., Smedby O., Landtblom A.M., Lundberg P., Larsson E.M. (2012). Synthetic MRI of the Brain in a Clinical Setting. Acta radiol..

[10] Tanenbaum L.N., Tsiouris A.J., Johnson A.N., Naidich T.P., DeLano M.C., Melhem E.R., Quarterman P., Parameswaran S.X., Shankaranarayanan A., Goyen M. (2017). Synthetic MRI for Clinical Neuroimaging: Results of the Magnetic Resonance Image Compilation (MAGiC) Prospective, Multicenter, Multireader Trial. Am. J. Neuroradiol..

[11] Di Giuliano F., Minosse S., Picchi E., Marfia G.A., Da Ros V., Muto M., Muto M., Pistolese C.A., Laghi A., Garaci F. (2020). Comparison between Synthetic and Conventional Magnetic Resonance Imaging in Patients with Multiple Sclerosis and Controls. Magn. Reson. Mater. Phys. Biol. Med..

[12] Di Giuliano F., Minosse S., Picchi E., Ferrazzoli V., Da Ros V., Muto M., Pistolese C.A., Garaci F., Floris R. (2021). Qualitative and Quantitative Analysis of 3D T1 Silent Imaging. Radiol. Medica.

[13] Chandarana H., Feng L., Block T.K., Rosenkrantz A.B., Lim R.P., Babb J.S., Sodickson D.K., Otazo R. (2013). Free-Breathing Contrast-Enhanced Multiphase MRI of the Liver Using a Combination of Compressed Sensing, Parallel Imaging, and Golden-Angle Radial Sampling. Investig. Radiol..

[14] Otazo R., Kim D., Axel L., Sodickson D.K. (2010). Combination of Compressed Sensing and Parallel Imaging for Highly Accelerated First-pass Cardiac Perfusion MRI. Magn. Reson. Med..

[15] Yoon J.K., Kim M.-J., Lee S. (2019). Compressed Sensing and Parallel Imaging for Double Hepatic Arterial Phase Acquisition in Gadoxetate-Enhanced Dynamic Liver Magnetic Resonance Imaging. Investig. Radiol..

[16] He M., Xu J., Sun Z., Wang S., Zhu L., Wang X., Wang J., Feng F., Xue H., Jin Z. (2020). Comparison and Evaluation of the Efficacy of Compressed SENSE (CS) and Gradient- and Spin-echo (GRASE) in Breath-hold (BH) Magnetic Resonance Cholangiopancreatography (MRCP). J. Magn. Reson. Imaging.

[17] Vranic J.E., Cross N.M., Wang Y., Hippe D.S., de Weerdt E., Mossa-Basha M. (2019). Compressed Sensing–Sensitivity Encoding (CS-SENSE) Accelerated Brain Imaging: Reduced Scan Time without Reduced Image Quality. Am. J. Neuroradiol..

[18] Sasi S D., Ramaniharan A.K., Bhattacharjee R., Gupta R.K., Saha I., Van Cauteren M., Shah T., Gopalakrishnan K., Gupta A., Singh A. (2020). Evaluating Feasibility of High Resolution T1-Perfusion MRI with Whole Brain Coverage Using Compressed SENSE: Application to Glioma Grading. Eur. J. Radiol..

[19] Meister R.L., Groth M., Jürgens J.H.W., Zhang S., Buhk J.H., Herrmann J. (2022). Compressed SENSE in Pediatric Brain Tumor MR Imaging. Clin. Neuroradiol..

[20] Cho S.J., Choi Y.J., Chung S.R., Lee J.H., Baek J.H. (2019). High-Resolution MRI Using Compressed Sensing-Sensitivity Encoding (CS-SENSE) for Patients with Suspected Neurovascular Compression Syndrome: Comparison with the Conventional SENSE Parallel Acquisition Technique. Clin. Radiol..

[21] Nagata S., Goshima S., Noda Y., Kawai N., Kajita K., Kawada H., Tanahashi Y., Matsuo M. (2019). Magnetic Resonance Cholangiopancreatography Using Optimized Integrated Combination with Parallel Imaging and Compressed Sensing Technique. Abdom. Radiol..

[22] Vasanawala S.S., Alley M.T., Hargreaves B.A., Barth R.A., Pauly J.M., Lustig M. (2010). Improved Pediatric MR Imaging with Compressed Sensing. Radiology.

[23] Liu F., Duan Y., Peterson B.S., Kangarlu A. (2012). Compressed Sensing MRI Combined with SENSE in Partial k -Space. Phys. Med. Biol..

[24] Mönch S., Sollmann N., Hock A., Zimmer C., Kirschke J.S., Hedderich D.M. (2020). Magnetic Resonance Imaging of the Brain Using Compressed Sensing—Quality Assessment in Daily Clinical Routine. Clin. Neuroradiol..

[25] Molnar U., Nikolov J., Nikolić O., Boban N., Subašić V., Till V. (2021). Diagnostic Quality Assessment of Compressed SENSE Accelerated Magnetic Resonance Images in Standard Neuroimaging Protocol: Choosing the Right Acceleration. Phys. Medica.

[26] Robson P.M., Grant A.K., Madhuranthakam A.J., Lattanzi R., Sodickson D.K., Mckenzie C.A. (2008). Comprehensive Quantification of Signal-to-Noise Ratio and *g*-Factor for Image-Based and *k*-Space-Based Parallel Imaging Reconstructions. Magn. Reson. Med..

[27] Reeder S.B., Wintersperger B.J., Dietrich O., Lanz T., Greiser A., Reiser M.F., Glazer G.M., Schoenberg S.O. (2005). Practical Approaches to the Evaluation of Signal-to-Noise Ratio Performance with Parallel Imaging: Application with Cardiac Imaging and a 32-Channel Cardiac Coil. Magn. Reson. Med..

[28] Aja-Fernández S., Vegas-Sánchez-Ferrero G., Tristán-Vega A. (2014). Noise Estimation in Parallel MRI: GRAPPA and SENSE. Magn. Reson. Imaging.

[29] Sartoretti E., Sartoretti T., Binkert C., Najafi A., Schwenk Á., Hinnen M., van Smoorenburg L., Eichenberger B., Sartoretti-Schefer S. (2019). Reduction of Procedure Times in Routine Clinical Practice with Compressed SENSE Magnetic Resonance Imaging Technique. PLoS ONE.

[30] Duan Y., Zhang J., Zhuo Z., Ding J., Ju R., Wang J., Ma T., Haller S., Liu Y., Liu Y. (2020). Accelerating Brain 3D T1-Weighted Turbo Field Echo MRI Using Compressed Sensing-Sensitivity Encoding (CS-SENSE). Eur. J. Radiol..

[31] Sartoretti T., Sartoretti E., van Smoorenburg L., Schwenk Á., Mannil M., Graf N., Binkert C.A., Wyss M., Sartoretti-Schefer S. (2020). Spiral 3-Dimensional T1-Weighted Turbo Field Echo: Increased Speed for Magnetization-Prepared Gradient Echo Brain Magnetic Resonance Imaging. Investig. Radiol..

[32] Okuchi S., Fushimi Y., Okada T., Yamamoto A., Okada T., Kikuchi T., Yoshida K., Miyamoto S., Togashi K. (2019). Visualization of Carotid Vessel Wall and Atherosclerotic Plaque: T1-SPACE vs. Compressed Sensing T1-SPACE. Eur. Radiol..

[33] Suh C.H., Jung S.C., Lee H.B., Cho S.J. (2019). High-Resolution Magnetic Resonance Imaging Using Compressed Sensing for Intracranial and Extracranial Arteries: Comparison with Conventional Parallel Imaging. Korean J. Radiol..

[34] Sartoretti T., Reischauer C., Sartoretti E., Binkert C., Najafi A., Sartoretti-Schefer S. (2018). Common Artefacts Encountered on Images Acquired with Combined Compressed Sensing and SENSE. Insights Imaging.

[35] Yang A.C., Kretzler M., Sudarski S., Gulani V., Seiberlich N. (2016). Sparse Reconstruction Techniques in Magnetic Resonance Imaging. Investig. Radiol..

[36] Sharma S.D., Fong C.L., Tzung B.S., Law M., Nayak K.S. (2013). Clinical Image Quality Assessment of Accelerated Magnetic Resonance Neuroimaging Using Compressed Sensing. Investig. Radiol..

