# Supplementary material for: Compressed SENSitivity Encoding (SENSE): Qualitative and Quantitative Analysis
Source: Diagnostics (Basel). 2024 Aug 5;14(15):1693. doi: 10.3390/diagnostics14151693 (PMC11311492; doi:10.3390/diagnostics14151693)
Supplement: Supplementary file 1 [file diagnostics-14-01693-s001.zip › diagnostics-3061890-supplementary.pdf]

Table S1. Qualitative subgroups analysis.

	Sequence	K	Group 1	K	Group 2	K	Group 3
Compressed-SENSE	3D FLAIR	0.42	Moderate	0.41	Moderate	0.58	Moderate
	T1	0.80	Good	0.30	Fair	0.23	Fair
	T2	0.47	Moderate	0.43	Moderate	0.54	Moderate
No Compressed-SENSE	3D FLAIR	0.60	Moderate	0.78	Good	0.65	Good
	T1	0.41	Moderate	0.43	Moderate	0.77	Good
	T2	0.40	Fair	0.48	Moderate	0.85	Very good

Table S2. Summary statistics of Contrast (C) of **Group 1** (age range 13-37).

		Compressed-SENSE			*No Compressed-SENSE*			
		Median	25th	75th	Median	25th	75th	p-value
FLAIR	GM-WM	0.11	0.03	0.19	0.10	0.03	0.16	0.225
	GM-CSF	0.62	0.53	0.65	0.69	0.61	0.75	0.001*
	WM-CSF	0.53	0.45	0.61	0.65	0.54	0.72	<0.001*
T1	GM-WM	-0.18	-0.25	-0.13	-0.20	-0.30	-0.14	0.039
	GM-CSF	0.68	0.64	0.72	0.64	0.56	0.68	0.011*
	WM-CSF	0.78	0.74	0.79	0.74	0.73	0.77	0.028*
T2	GM-WM	0.12	0.06	0.19	0.12	0.05	0.17	0.438
	GM-CSF	-0.51	-0.54	-0.47	-0.38	-0.42	-0.32	<0.001*
	WM-CSF	-0.59	-0.62	-0.57	-0.47	-0.50	-0.43	<0.001*

Data are expressed as median, 25th and 75th percentile. p-values refer to the Wilcoxon test. Statistically significant correlations for *p < 0.05. White matter (WM), grey matter (GM) and cerebrospinal fluid (CSF).

Table S3. Summary statistics of Contrast-to-Noise Ratio (CNR) **Group 1** (age range 13-37).

		Compressed-SENSE			*No Compressed-SENSE*			
		Median	25th	75th	Median	25th	75th	p-value
FLAIR	GM-WM	2.69	1.05	5.27	2.60	0.66	4.53	0.365
	GM-CSF	11.95	9.28	13.90	12.70	10.42	14.91	0.392
	WM-CSF	9.08	5.91	11.14	9.87	7.93	11.67	0.178
T1	GM-WM	-9.41	-12.41	-7.13	-8.99	-11.84	-6.41	0.262
	GM-CSF	17.03	14.11	20.60	12.82	9.88	16.29	0.001*
	WM-CSF	26.67	24.10	29.35	22.24	18.84	25.79	0.002*
T2	GM-WM	4.31	2.26	7.33	4.91	2.01	7.81	0.641
	GM-CSF	-43.05	-50.97	-34.17	-28.57	-33.62	-20.84	<0.001*
	WM-CSF	-46.39	-58.74	-41.24	-34.36	-41.36	-23.15	<0.001*

Data are expressed as median, 25th and 75th percentile. p-values refer to the Wilcoxon test. Statistically significant correlations for *p < 0.05. White matter (WM), grey matter (GM) and cerebrospinal fluid (CSF).

Table S4. Summary statistics of Signal-to-Noise Ratio (SNR) **Group 1** (age range 13-37).

		Compressed- SENSE			*No Compressed-SENSE*			
		Median	25th	75th	Median	25th	75th	p-value
FLAIR	FC	19.64	15.50	23.80	18.68	17.30	20.99	0.717
	Ge	11.69	8.86	13.48	12.27	11.33	13.04	0.818
	CSF	3.81	3.37	4.44	2.76	2.34	3.22	0.011*
	Sp	11.86	9.70	15.73	11.75	10.99	13.26	0.921
	CS	14.46	12.58	18.81	15.15	13.65	17.20	0.869
	OC	15.63	12.43	17.14	14.72	13.89	16.20	0.575
	Th	13.57	11.54	16.12	13.93	11.97	15.64	0.921
T1	FC	17.52	16.31	19.02	13.45	11.64	16.71	0.023*
	Ge	31.38	27.74	34.02	27.34	21.80	31.51	0.156
	CSF	4.18	3.39	4.67	3.92	2.92	4.33	0.339
	Sp	30.47	28.27	34.78	26.95	22.24	30.94	0.223
	CS	31.08	26.81	32.07	25.76	22.15	31.61	0.081
	OC	21.82	18.67	24.38	17.81	15.25	19.75	0.052
	Th	24.07	21.57	26.20	19.78	17.45	25.09	0.121
T2	FC	24.55	20.94	29.23	30.24	20.37	36.10	0.199
	Ge	14.36	12.63	16.38	19.22	14.23	21.13	0.093
	CSF	63.11	54.27	79.84	55.90	36.06	64.97	0.177
	Sp	16.33	13.92	18.15	20.48	12.89	22.76	0.307
	CS	17.67	16.34	21.31	23.27	15.41	25.96	0.177
	OC	18.75	14.82	22.63	24.16	14.54	25.74	0.138
	Th	20.34	17.11	23.67	25.75	16.36	27.21	0.307

Data are expressed as median, 25th and 75th percentile. p-values refer to the Wilcoxon test. Statistically significant correlations for *p < 0.05. White matter (WM), grey matter (GM) and cerebrospinal fluid (CSF). Frontal cortex (FC), genu of corpus callosum (Ge), splenium of corpus callosum (Sp), centrum semiovale (CS), occipital cortex (OC), thalami (Th).

Table S5. Summary statistics of Contrast (C) of **Group 2** (age range 38-62).

		Compressed-SENSE			*No Compressed-SENSE*			
		Median	25th	75th	Median	25th	75th	p-value
FLAIR	GM-WM	0.07	0.00	0.15	0.09	0.01	0.16	0.277
	GM-CSF	0.63	0.56	0.71	0.76	0.70	0.80	<0.001*
	WM-CSF	0.58	0.52	0.66	0.73	0.63	0.76	<0.001*
T1	GM-WM	-0.19	-0.24	-0.13	-0.18	-0.25	-0.13	0.448
	GM-CSF	0.68	0.63	0.73	0.66	0.61	0.71	0.190
	WM-CSF	0.77	0.72	0.80	0.76	0.72	0.78	0.092
T2	GM-WM	0.10	0.05	0.16	0.10	0.05	0.17	0.728
	GM-CSF	-0.52	-0.55	-0.47	-0.40	-0.44	-0.34	<0.001*
	WM-CSF	-0.59	-0.62	-0.55	-0.48	-0.51	-0.45	<0.001*

Data are expressed as median, 25th and 75th percentile. p-values refer to the Wilcoxon test. Statistically significant correlations for *p < 0.05. White matter (WM), grey matter (GM) and cerebrospinal fluid (CSF).

Table S6. Summary statistics of Contrast-to-Noise Ratio (CNR) of **Group 2** (age range 38-62).

		Compressed-SENSE			*No Compressed-SENSE*			
		Median	25th	75th	Median	25th	75th	p-value
FLAIR	GM-WM	1.83	-0.16	4.74	2.35	0.43	4.58	0.429
	GM-CSF	11.73	8.73	15.20	13.28	10.55	15.75	0.100
	WM-CSF	9.56	7.47	12.61	11.02	9.01	12.66	0.109
T1	GM-WM	-9.31	-11.93	-6.54	-8.83	-12.41	-6.25	0.596
	GM-CSF	16.73	12.81	20.43	16.43	12.54	21.04	0.953
	WM-CSF	25.24	22.13	30.95	25.96	21.91	32.08	0.878
T2	GM-WM	3.65	1.33	6.20	4.73	2.47	8.76	0.001*
	GM-CSF	-42.23	-50.97	-33.69	-36.29	-43.89	-29.06	0.001*
	WM-CSF	-46.46	-56.21	-36.89	-42.41	-47.58	-34.63	0.010*

Data are expressed as median, 25th and 75th percentile. p-values refer to the Wilcoxon test. Statistically significant correlations for $*p < 0.05$. White matter (WM), grey matter (GM) and cerebrospinal fluid (CSF).

Table S7. Summary statistics of Signal-to-Noise Ratio (SNR) **Group 2** (age range 38-62).

		Compressed- SENSE			*No Compressed-SENSE*			
		Median	25th	75th	Median	25th	75th	p-value
FLAIR	FC	19.47	16.43	24.20	18.39	16.18	22.10	0.374
	Ge	12.43	10.67	14.68	12.99	10.47	14.48	0.913
	CSF	3.25	2.76	4.29	1.97	1.74	2.42	<0.001*
	Sp	11.45	9.79	15.52	12.00	9.59	13.72	0.798
	CS	15.10	13.47	18.90	15.19	13.97	18.36	0.724
	OC	12.90	12.09	17.87	15.23	13.58	16.47	0.401
	Th	12.93	10.78	16.67	12.99	12.03	15.93	0.990
T1	FC	18.27	14.07	21.33	16.49	15.07	21.24	0.913
	Ge	31.31	24.27	35.23	30.03	24.79	37.57	0.952
	CSF	3.79	3.24	4.55	4.22	3.63	4.92	0.162
	Sp	28.59	24.85	35.89	31.13	25.70	36.11	0.932
	CS	29.64	25.30	34.52	30.28	25.95	37.07	0.780
	OC	20.63	16.72	24.56	20.65	16.89	25.53	0.952
	Th	22.87	19.73	28.19	22.93	20.65	29.28	0.798
T2	FC	24.57	20.41	27.34	32.92	28.34	36.15	<0.001*
	Ge	15.87	12.70	18.14	21.30	17.72	23.11	<0.001*
	CSF	63.11	50.70	75.09	64.88	54.24	74.32	0.990
	Sp	17.28	13.23	18.63	21.30	19.25	23.89	0.001*
	CS	18.60	15.36	21.23	24.45	21.05	26.91	<0.001*
	OC	19.01	15.08	19.93	24.64	22.63	28.01	<0.001*
	Th	20.75	16.28	22.86	25.90	23.74	29.16	<0.001*

Data are expressed as median, 25th and 75th percentile. p-values refer to the Wilcoxon test. Statistically significant correlations for $*p < 0.05$. White matter (WM), grey matter (GM) and cerebrospinal fluid (CSF). Frontal cortex (FC), genu of corpus callosum (Ge), splenium of corpus callosum (Sp), centrum semiovale (CS), occipital cortex (OC), thalami (Th).

Table S8. Summary statistics of Contrast (C) **Group 3** (age range 63-86).

		Compressed-SENSE			***No Compressed-SENSE***			
		Median	25th	75th	Median	25th	75th	p-value
FLAIR	GM-WM	0.09	0.00	0.17	0.07	0.00	0.14	0.021
	GM-CSF	0.64	0.59	0.69	0.80	0.74	0.83	<0.001*
	WM-CSF	0.60	0.53	0.63	0.77	0.70	0.82	<0.001*
T1	GM-WM	-0.17	-0.22	-0.12	-0.19	-0.25	-0.13	0.004*
	GM-CSF	0.68	0.64	0.71	0.64	0.59	0.69	<0.001*
	WM-CSF	0.76	0.74	0.78	0.75	0.71	0.77	<0.001*
T2	GM-WM	0.10	0.04	0.16	0.10	0.04	0.16	0.774
	GM-CSF	-0.53	-0.56	-0.48	-0.40	-0.43	-0.33	<0.001*
	WM-CSF	-0.59	-0.62	-0.56	-0.48	-0.51	-0.43	<0.001*

Data are expressed as median, 25th and 75th percentile. p-values refer to the Wilcoxon test. Statistically significant correlations for *p < 0.05. White matter (WM), grey matter (GM) and cerebrospinal fluid (CSF).

Table S9. Summary statistics of Contrast-to-Noise Ratio (CNR) **Group 3** (age range 63-86).

		Compressed-SENSE			***No Compressed-SENSE***			
		Median	25th	75th	Median	25th	75th	p-value
FLAIR	GM-WM	2.460	0.088	4.608	1.737	0.086	3.463	0.024*
	GM-CSF	11.281	8.874	14.163	12.587	9.564	15.462	0.017*
	WM-CSF	8.578	6.871	11.275	10.754	8.160	13.335	<0.001*
T1	GM-WM	-8.853	-11.882	-5.948	-8.743	-11.755	-6.061	0.829
	GM-CSF	17.346	14.324	20.734	15.027	12.025	18.494	0.001*
	WM-CSF	25.209	22.105	31.801	24.601	19.511	28.427	0.022*
T2	GM-WM	3.804	1.572	6.161	4.649	1.658	7.859	0.006*
	GM-CSF	-44.202	-52.574	-37.497	-33.210	-39.540	-27.702	<0.001*
	WM-CSF	-47.465	-59.110	-40.825	-37.445	-45.243	-31.456	<0.001*

Data are expressed as median, 25th and 75th percentile. p-values refer to the Wilcoxon test. Statistically significant correlations for *p < 0.05. White matter (WM), grey matter (GM) and cerebrospinal fluid (CSF).

Table S10. Summary statistics of Signal-to-Noise Ratio (SNR) **Group 3** (age range 63-86).

		Compressed- SENSE			***No Compressed-SENSE***			
		Median	25th	75th	Median	25th	75th	p-value
FLAIR	FC	18.20	15.11	19.34	17.90	13.88	19.51	0.269
	Ge	11.32	9.86	12.55	12.27	9.48	14.78	0.282
	CSF	3.10	2.92	3.66	1.62	1.28	2.06	<0.001*
	Sp	10.26	8.97	12.57	10.70	8.71	12.81	0.695
	CS	14.79	12.97	17.19	15.01	11.88	17.47	0.600
	OC	13.88	11.68	16.36	14.24	11.10	16.51	0.795
	Th	12.15	10.58	14.65	12.81	10.37	14.97	0.972
T1	FC	19.36	14.59	22.69	17.04	14.13	19.44	0.072
	Ge	28.86	23.13	36.38	28.40	23.05	32.82	0.436
	CSF	4.18	3.49	4.98	4.20	3.76	5.06	0.460
	Sp	29.91	26.09	36.07	29.74	24.51	33.50	0.316
	CS	28.92	26.13	36.85	29.31	23.90	31.74	0.128
	OC	20.67	17.95	25.76	19.63	17.77	22.66	0.066
	Th	23.97	20.32	28.71	22.24	18.51	26.25	0.256
T2	FC	22.66	20.04	27.69	29.34	26.91	37.96	<0.001*
	Ge	15.86	13.93	18.07	20.53	17.39	24.84	<0.001*
	CSF	64.21	54.15	78.13	60.28	51.17	72.69	0.084
	Sp	15.57	13.07	19.68	19.62	15.96	23.55	0.003*
	CS	18.57	16.82	21.80	23.51	20.94	29.17	<0.001*
	OC	18.14	15.55	21.70	23.00	18.59	28.47	<0.001*
	Th	20.96	17.54	24.29	25.10	22.05	31.15	0.002*

Data are expressed as median, 25th and 75th percentile. p-values refer to the Wilcoxon test. Statistically significant correlations for *$p < 0.05$. White matter (WM), grey matter (GM) and cerebrospinal fluid (CSF). Frontal cortex (FC), genu of corpus callosum (Ge), splenium of corpus callosum (Sp), centrum semiovale (CS), occipital cortex (OC), thalami (Th).
